# From Phenotypes to Genotypes: Enhancing the Identification of *Cymbidium* Species with DNA Barcoding

**DOI:** 10.3390/plants14040619

**Published:** 2025-02-18

**Authors:** Yaonan Peng, Yao Chen, Hongfan Ding, Xiangdong Liu, Fuxiang Cao, Lu Xu

**Affiliations:** 1 College of Horticulture, Hunan Agricultural University, Changsha 410128, China; pengyaonan2022@163.com (Y.P.); m13201018828@163.com (Y.C.); 19896267791@163.com (H.D.); m15116158531_1@163.com (X.L.); 2 Hunan Mid-Subtropical Quality Plant Breeding and Utilization Engineering Technology Research Center, Changsha 410128, China; 3 Yuelushan Laboratory, Changsha 410128, China

**Keywords:** *Cymbidium*, phenotypic diversity, DNA barcoding, classification identification, affiliation

## Abstract

The genus *Cymbidium*, with its intricate floral elements, pronounced endemicity, and patchy distribution, evolves a rich diversity of morphological forms and a wide variety of species while causing an indistinctness in the classification of its species. To elucidate the phylogenetic relationships among *Cymbidium* species and enhance their taxonomic classification by DNA barcoding, this study conducted amplification and sequence results of nuclear (ITS) and chloroplast genes (*matK*, *rbcL*, *trnL-F*, *psbA-trnH*) with phenotypic genetic diversity analysis, genetic distance analysis, and phylogenetic analysis from 48 samples of *Cymbidium* species. The comparison of genetic distance variations showed that *psbA-trnH*, ITS + *psbA-trnH*, and ITS + *matK* + *psbA-trnH* exhibit minimal overlap and significant genetic variation within *Cymbidium* species. The phylogenetic analysis indicated that the combination, ITS + *matK* + *psbA-trnH*, has the highest identification rate. Notably, both the phylogenetic analysis and the genetic diversity analysis of phenotypic traits consistently indicated a clear divergence between epiphytic and terrestrial orchids, with epiphytic orchids forming a distinct clade. This provides reference evidence for studying the ecological adaptations and evolutionary differences between epiphytic and terrestrial orchids, as well as a scientific basis for the classification and identification, germplasm conservation, resource utilization, and phylogenetic evolution of orchids.

## 1. Introduction

*Cymbidium* Sw., a member of the Orchidaceae family within the Epidendroideae subfamily, includes perennial herbs predominantly found in the tropical and subtropical regions of Asia, extending southward to Australia [1]. It stands as one of the most prolific flowering families globally. The genus *Cymbidium* occupies a leading position in orchid plants, with its scientific, economic, cultural, and social values being of paramount importance. Notably, the terrestrial species (such as *C. goeringii*, *C. faberi*, *C. ensifolium*, *C. kanran*, and *C. sinense*), colloquially known as ‘Guolan’ in China, have been cultivated in the country for over two millennia [2]. Since 1981, scholars have initiated the establishment of orchid germplasm conservation nurseries in Zhejiang Province, with subsequent centers and nature reserves being set up in various provinces such as Guangxi, Jiangsu, and Guangdong, emphasizing the importance of enhancing and safeguarding orchid germplasm resources [3]. This indicates that the preservation and utilization of orchid germplasm resources in China have officially commenced. However, the widespread natural hybridization phenomenon of *Cymbidium* species enhances the diversity of morphological features but makes its classification ambiguous. Moreover, the nomenclature system for *Cymbidium* resources lacks standardization, leading to frequent issues with synonyms and homonyms [4]. The confusion between Latin and Chinese names, coupled with the outdated methods of information resource management, impedes the conservation, exchange, utilization, and innovation of *Cymbidium* germplasm resources. These factors exacerbate the difficulties in research classification and the study of genetic diversity within *Cymbidium* species [5].

The infrageneric classification of *Cymbidium* was initially based on the classification system proposed by Schlechter, which divided the genus into eight sections [6]. Scholars initially categorized the genus *Cymbidium* into three subgenera, such as subgenus *Cymbidium*, subgenus *Cyperorchis*, and subgenus *Jensoa* [7]. Subsequently, Puy and Cribb [8] and Liu et al. [9] made further discoveries regarding the classification of the genus and conducted supplementary research and refinements. However, morphological characteristics and their statistical descriptions are easily influenced by environmental variability, making it challenging to distinguish *Cymbidium* species solely based on phenotypic traits. Therefore, utilizing more stable and concise methods for the identification and classification of *Cymbidium* species is extremely necessary.

DNA barcoding is a method of molecular identification that employs short, standardized DNA sequences to swiftly ascertain the species of biological specimens [10,11,12,13]. In 2003, Hebert first formally proposed the concept of DNA barcoding at the first International Conference on the Barcoding of Life (CBOL), introducing molecular biology techniques into the broader realm of biological classification [14] and announcing it as an essential tool for the identification of species across the globe. The Sloan Gene Society (2004) established a consortium for the CBOL to develop a standard barcode process and a comprehensive DNA barcode database and gradually extend DNA barcoding research to unknown species and to develop a global standard for species identification [15]. At the Third International Conference on DNA Barcoding in 2009, *rbcL* and *matK* were proposed as core barcodes for terrestrial plants, with the rapidly evolving ITS and *psbA-trnH* sequences suggested as supplementary barcodes for plant identification [16]. The Chinese Plant Barcoding Consortium assessed the identification ability of *psbA-trnH*, ITS/ITS2 and *rbcL* + *matK* sequences or combinations in 2011. The results showed that the ITS/ITS2 sequence could serve as the core barcode for seed plants, with *psbA-trnH* emerging as a candidate barcode sequence [11]. In addition, previous studies have shown that a complete chloroplast genome can serve as an effective tool for identifying *Cymbidium* species and resolving their phylogenetic relationships [17,18,19]. Despite some progress in these studies, the phylogenetic relationships among many *Cymbidium* species remain controversial [20,21,22]. Therefore, more effective molecular techniques are needed for further research on orchid species. As a form of digital information, DNA barcoding technology holds significant value in taxonomy with its accuracy, richness, and unique repeatability [12,23,24]. With the rapid advancement of biotechnology, sequencing reactions are becoming more accessible and cost-effective, facilitating the comprehensive construction of public sequence databases. Consequently, DNA barcoding, with its speed and efficiency, is becoming increasingly practical [25]. Its applications extend beyond taxonomy to include evolutionary ecology, food quality and safety, forensic evidence, pharmacology, and other fields of identification.

The application of DNA barcoding technology has greatly enriched the genetic information of *Cymbidium* species, aiding in the exploration of plant morphological evolution caused by genetic mutations and variations. Therefore, in order to resolve the taxonomic ambiguities caused by the rich and diverse phenotypic variations, genetic variations, and taxonomic confusion in *Cymbidium*, this study employed five single-DNA-fragment barcodes and four combined barcodes to conduct phylogenetic analysis on 48 plant materials of *Cymbidium*. Concurrently, combined with morphological classification, we aim to identify superior and more precise DNA sequence markers for the classification of *Cymbidium* species and comprehensively explore the affiliation and potential divergence of closely related *Cymbidium* species across different life forms. This study aspires to tackle theoretical challenges in the classification of *Cymbidium* species, lay a foundation for clarifying the genetic variation of *Cymbidium*, and provide basic technical support for regulating and managing orchids that can be used in legal trade, as well as for accurately determining the geographical origin of unknown specimens in illegal trade.

## 2. Results

### 2.1. Analysis of Amplification Success Rate and Sequence Characteristics

We initially employed five barcode sequences to amplify and sequence 48 samples of *Cymbidium* species. The findings demonstrated a 100% success rate in amplifying the five DNA barcodes (Table 1), along with a 100% success rate in sequencing *matK* and *rbcL* genes. Additionally, the ITS, *psbA-trnH*, and *trnL-F* genes exhibited a sequencing success rate of 95.8%, resulting in obtaining ideal sequences from 46 samples. These results indicate that the amplification and sequencing outcomes of these five barcodes were satisfactory, rendering them suitable for subsequent research on *Cymbidium* using DNA barcoding.

Among the single sequences, the analysis of each barcode sequence (Table 2) revealed that the length of sequence alignment was ordered as follows: ITS > *psbA-trnH* > *matK* > *trnL-F* > *rbcL*. The GC content, ranked from highest to lowest, was ITS > *rbcL* > *rnL-F* > *psbA-trnH* > *matK*. Considering the percentage of each site relative to the total length, ITS exhibited the greatest variability, while *rbcL* showed the highest level of conservation. In summary, ITS has the potential to become the DNA barcode of *Cymbidium* species.

Among the combination sequences, the results (Table 3) indicated that the ITS +* matK* + *psbA-trnH* sequence yielded the longest sequence alignment length, and the ITS +* psbA-trnH* sequence resulted in the shortest sequence alignment length. The GC content, when ranked from highest to lowest, was as follows: ITS +* psbA-trnH* > ITS +* matK* > *psbA-trnH *+ ITS +* matK* > *matK* + *psbA-trnH*. Considering the percentage of variation sites to the length, the highest proportion was ITS +* psbA-trnH* (17.8 %), followed by ITS +* matK* (17.7 %), with a very minimal difference between them. In terms of the percentage of conserved sites to the length, the proportion of *matK* +* psbA-trnH* was the highest (87.8 %), followed by ITS +* matK* + *psbA-trnH* (83.8 %). Based on the above information, it was concluded that ITS +* psbA-trnH* exhibited the greatest variability, while the *matK* + *psbA-trnH* showed the strongest conservation.

### 2.2. Analysis of Barcoding Gap

The ITS, *matK*, *psbA-trnH*, *rbcL*, and *trnL-F* all produced correct sequencing signals and were used for resolution analyses. The results of genetic distance for the five single-segment barcodes (Table 4) indicated that the average intraspecific genetic distance, from largest to smallest, was ordered as ITS > psbA-trnH > *matK* > *trnL-F* > *rbcL*, and the average interspecific genetic distance followed the same order: ITS > *psbA-trnH* > *matK* > *trnL-F* > *rbcL*. The smallest genetic distance, both interspecific and intraspecific, was observed for *rbcL*. The barcode ITS exhibited larger interspecific and intraspecific genetic distances, and the average interspecific genetic distance for each barcode was greater than the average intraspecific genetic distance. Based on the above information, ITS displayed the highest level of genetic variation, followed by *psbA-trnH*, while *rbcL* showed the lowest level of genetic variation.

Among the combined barcode genetic distance (Table 5), the findings revealed that the order of average intraspecific genetic distance was ITS + *psbA-trnH* > ITS + *matK* > ITS + *matK* + *psbA-trnH* > *matK* + *psbA-trnH*. The order of average interspecific genetic distance was ITS + *psbA-trnH* > ITS + *matK* > ITS + *matK* + *psbA-trnH* > *matK* + *psbA-trnH*. The largest interspecific and intraspecific genetic distance was observed for the ITS + *psbA-trnH*, with the smallest one, *matK* + *psbA-trnH*. The average interspecific genetic distance of barcodes in each combination was greater than the average intraspecific genetic distance. Based on the above information, it is evident that ITS + *psbA-trnH* exhibited a relatively large variation in intraspecific genetic distance, followed by ITS + *matK*.

**Figure 1:**
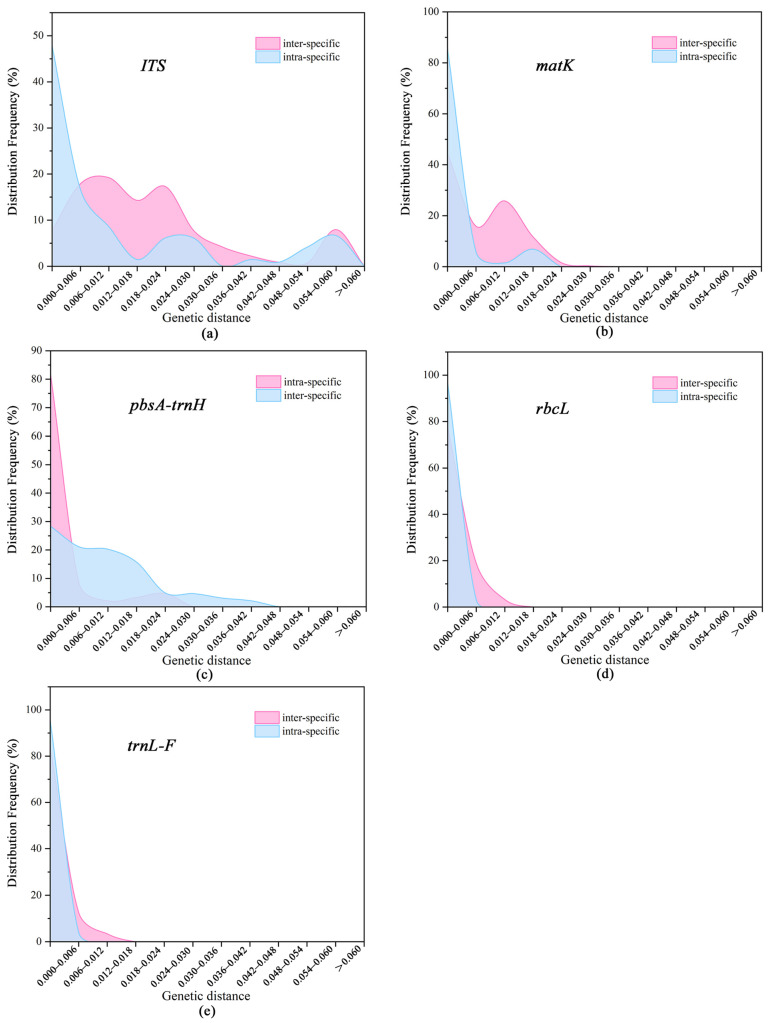
Barcoding gap distribution of five monolithic segments. (**a**) ITS; (**b**) *matK*; (**c**) *psbA-trnH*; (**d**) rbcL; and (**e**) *trnL-F*.

In this study, we plotted the distributions of interspecific and intraspecific genetic distances for five barcode sequences to evaluate the barcoding gap among different barcodes. The results showed that there is a certain overlap in the distribution of interspecific and intraspecific genetic distances of DNA barcodes in *Cymbidium* species, but the overlap of ITS and *psbA-trnH* barcodes is minimal and skewed towards the extremes compared to other barcodes, with the *psbA-trnH* sequence showing the least overlap (Figure 1). In summary, *psbA-trnH* and ITS sequences are relatively suitable for the identification of *Cymbidium* species.

**Figure 2:**
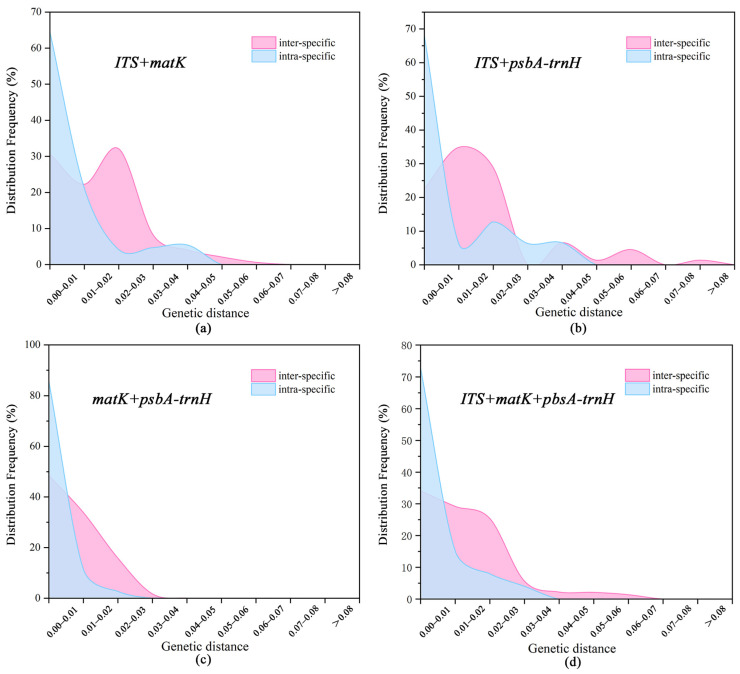
Barcoding gap distribution of four DNA barcode combination sequences. (**a**) ITS + *matK*; (**b**) ITS + *psbA-trnH*; (**c**) *matK* + *psbA-trnH*; and (**d**) ITS + *matK *+ *psbA-trnH*.

The genetic distance distribution plots of the four combined barcodes reveal that there are certain overlaps in the genetic distance distributions between intraspecific and interspecific barcodes for these combined barcodes (Figure 2). However, the variations in intraspecific genetic distance are primarily concentrated on the lower end of the value spectrum, while the variations in interspecific genetic distance are predominantly on the higher end. Among them, the ITS + *psbA-trnH* and ITS + *matK *+ *psbA-trnH* barcode combinations exhibit a trend of bidirectional dispersion in their distribution, with less overlap compared to other barcode combinations, which could become a focus of subsequent research.

### 2.3. Phylogenetic Analysis

To assess the genetic diversity of the *Cymbidium* species, a phylogenetic tree was constructed using different barcode regions and analyzed using the best match and best close match methods. In terms of the single-barcode identification rate, the ITS region exhibited the highest success rate at 41.66 %, followed by *psbA-trnH* at 41.30 %, and rbcL with the lowest value of 4.16 %. Combinations of two regions, such as ITS + *psbA-trnH*, demonstrated the lowest success rate of species discrimination at 43.47%. On the other hand, the highest species discrimination success was observed in combinations of ITS + *matK* + *psbA-trnH*, which showed a success rate of 55.55% (Table S1), followed by *matK* + *psbA-trnH* at 52.17%. The combination barcodes yielded a higher identification rate than individual barcodes due to the low level of variation in the *rbcL* and *trnL-F* region.

**Figure 3:**
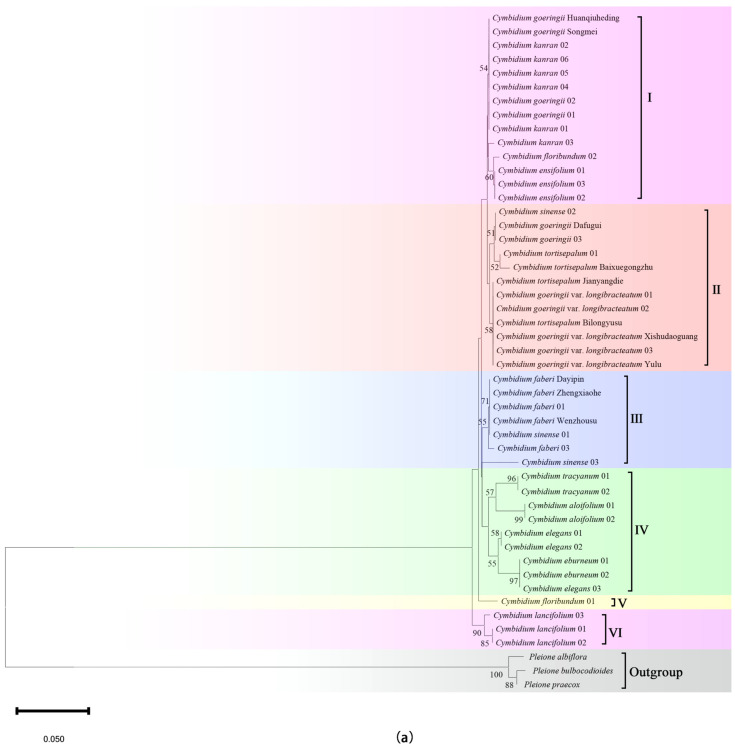

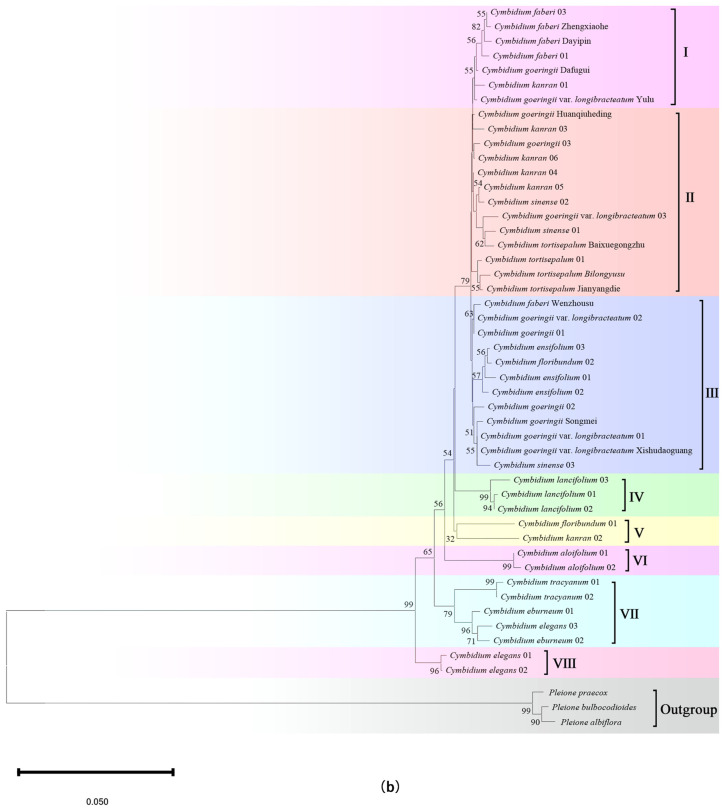
Phylogenetic tree of some *Cymbidium* species based on *psbA-trnH* and *matK* + *psbA-trnH*. Numbers above the branches indicate bootstrap (BS ≥ 50) values. (**a**) *psbA-trnH*, all *Cymbidium* species were classified into five primary clades: I–VI. (**b**) *matK* + *psbA-trnH*, all *Cymbidium* species were classified into five primary clades: I–VIII. The outgroup was represented by three species from *Pleione* D. Don.

Incorporating both sequence feature analysis and genetic distance analysis, the neighbor-joining phylogenetic tree was constructed based on *psbA-trnH* of the single-fragment barcodes, *matK* + *psbA-trnH* and ITS + *matK* + *psbA-trnH* of the combined barcodes to explore the taxonomic and phylogenetic relationships among *Cymbidium* species. The phylogenetic analysis (Figure 3) showed that all the *Cymbidium* species were classified into six primary clusters. All the species belonging to the sect. *Jensoa* of the subgenus *Jensoa* were grouped together. Most varieties of *C*. *goeringii* are clustered together, while *C*. *faberi* and its varieties are clustered into a separate clade. Four epiphytic orchids (*C. tracyanum*, *C. aloifolium*, *C. eburneum*, and *C. elegans*) were clustered into one category, and the terrestrial plant, *C. lancifolium*, is also categorized separately. The combination method of using two barcodes yielded similar results to the individual barcode approach, which revealed that all the *Cymbidium* species were classified into eight primary clusters.

**Figure 4:**
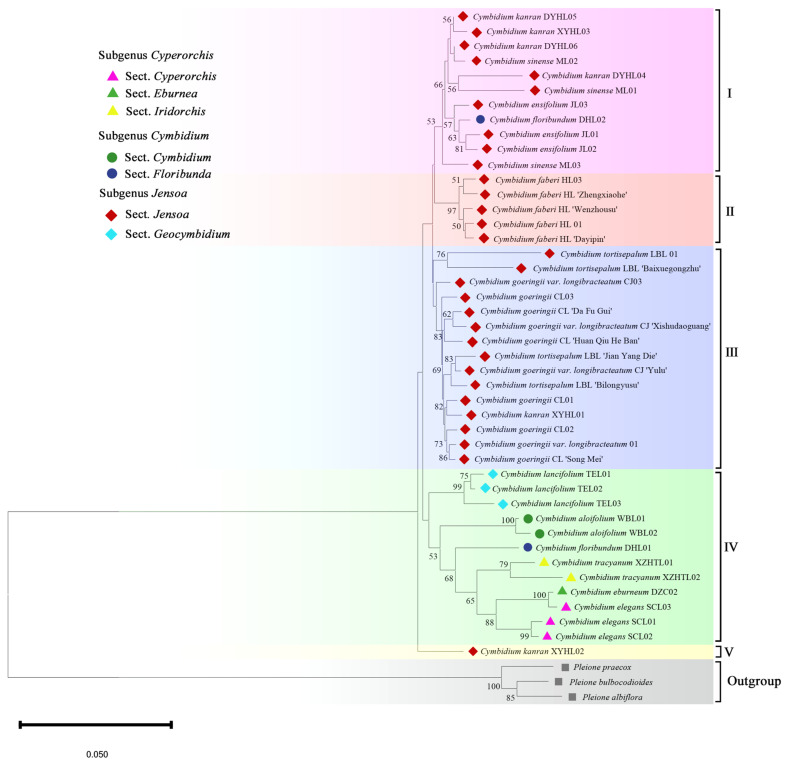
Phylogenetic tree of some *Cymbidium* species based on combined barcodes (ITS + *matK* + *psbA*-*trnH*). Numbers above the branches indicate bootstrap (BS ≥ 50) values. All *Cymbidium* species were classified into five primary clades: I–V. The outgroup was represented by three species from *Pleione* D. Don.

The phylogenetic tree, based on ITS + *matK* +* psbA-trnH* of three barcode combinations (Figure 4), reveals that all species within *Cymbidium* were categorized into seven primary clusters. Clade I comprised 11 species, which included one species from sect. *Floribunda* of the subgenus *Cymbidium*, while all the remaining species belong to the sect. *Jensoa* of the subgenus *Jensoa.* All species and varieties of *C. faberi* from the sect. *Jensoa* of the subgenus *Jensoa* were clustered in Clade II. All species of *C. goeringii*, some varieties of *C. tortisepalum*, and the variety of *C. goeringii* known as ‘Chunjian’, along with its varieties, together form Clade III, which is the most species-rich taxonomic group. The Clade IV was the most complex, with 12 species, including six from the subgenus *Cyperorchis* (three species from sect. *Cyperorchis*, one species from sect. *Eburnea*, and two species from sect. *Iridorchis*), one species from the subgenus *Cymbidium* (*C. floribundum* from sect. *Floribunda*), and three species from sect. *Geocymbidium* of the subgenus *Jensoa*. Clade Ⅴ was composed of *C. kanran* from the sect. *Jensoa* of the subgenus *Jensoa*.

The phylogenetic analysis showed that unsupported relationships were primarily found within the sect. *Jensoa* of the subgenus *Jensoa* and were often associated with low levels of sequence variation, with the bootstrap value above 50%. The two accessions of the epiphytic orchid (*C. floribundum*) appear in different clades. All epiphytic orchids were clustered in Clade IV, and the terrestrial *C. lancifolium* was also grouped within this clade. Through the analysis of the phylogenetic tree, we have discovered that the *Cymbidium* species with the same life forms exhibit closer affiliation. By integrating barcode sequence characteristics and genetic distance analysis, it was revealed that employing the combined barcode (ITS + *matK* + *psbA-trnH*) technique significantly improves the species identification rate and classification efficiency of *Cymbidium* species. To delve deeper into the taxonomic relationships among *Cymbidium* species, we have also conducted an analysis of the genetic diversity associated with phenotypic traits.

### 2.4. Genetic Diversity Analysis of Phenotypic Characters in Orchid Germplasm Resources

#### 2.4.1. Diversity Analysis of Quantitative Traits

We analyzed the variation of 11 quantitative traits of *Cymbidium* species and observed that the degree of diversity of different quantitative traits was also different. The coefficient of variation of 11 quantitative traits ranged from 28.2% to 128.9%, demonstrating a considerable degree of trait diversity (Table S2). Among them, the trait with the highest coefficient of variation was the number of flowers in the flower bract, followed by leaf width and the height of scape, indicating that these three traits exhibit high polymorphism. The petal width had the lowest coefficient of variation, followed by sepal length, indicating that these two characters were relatively stable. The average coefficient of variation of other quantitative traits was 36.8%, which was relatively concentrated. These findings reveal a rich genetic variation in phenotypic traits among different varieties of *Cymbidium* species.

The correlation analysis of 11 quantitative traits among orchids revealed that 21 pairs of traits exhibited significant correlations, with all being positive correlations (Table S3). Notably, eight pairs demonstrated significant positive correlations. The height of flower scape, length of leaf, number of flowers, and number of leaves all correlated significantly, indicating a reflection of the plant’s robustness. In contrast, leaf width, sepal length, sepal width, and petal width showed negative correlations. The most substantial correlation was found between flower length and labellum length, while the least significant correlation was detected between the number of flowers and sepal length. Significant correlations were also identified between the length and width of the sepal, the length and width of the labellum, and the length of the petal, suggesting a close interrelation between these floral structures.

#### 2.4.2. Diversity Evaluation of Quality Traits

We observed 16 quality traits of *Cymbidium* species phenotypes, including 2 leaf appearance traits, 13 petal appearance traits, and 1 pseudobulb trait. There were 52 variation types among the 16 quality traits, with an average of 3.2 variation types per trait. The Shannon–Wiener index ranged from 0.22 to 2.20 (Table S4), with an average of 1.14, among which the main color variation of the middle sepal was the highest, and the leaf tip pattern variation was the lowest. In the leaf appearance traits of *Cymbidium* species, the tip shape of most varieties is sharp, and the edge of the leaf is slightly serrated. The main color phenotype of the middle sepal was the most abundant, including white, green, yellow, red, purple, and brown, followed by the main color of the middle sepal and the middle petal. Regarding the labellum flaps, the shape and color of the lobes in the labellum lobe of most tested varieties were triangular and pale yellow. In terms of flower spots and stripes, most of the varieties to be tested had streaked sepals without stripes, petals with streaks without stripes, and labellum petals with spots without stripes. Among the characteristics of pseudobulb size, as epiphytic orchids, *C. floribundum*, *C. aloifolium*, *C. elegans*, and *C. tracyanum* have obviously larger pseudobulbs than those of other terrestrial orchids, which aligns with the characteristics of common epiphytic and terrestrial pseudobulbs.

#### 2.4.3. Principal Component Analysis of Phenotypic Traits

**Figure 5:**
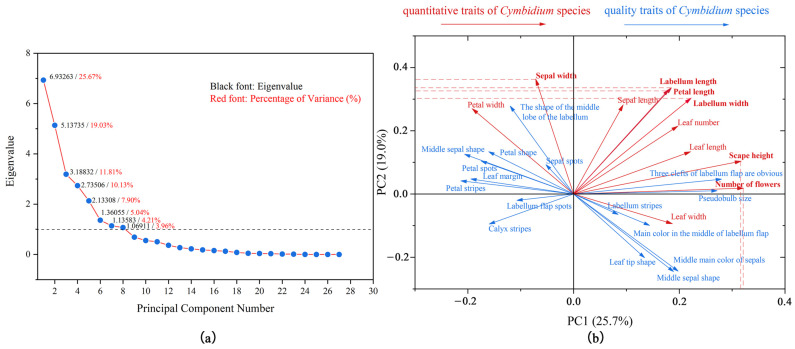
The principal component analysis of 27 phenotypic trait indicators among *Cymbidium* species. (**a**). The scree plot from principal component analysis. Includes the eigenvalues and contribution rates of each component. (**b**). The loading plot from principal component analysis. Includes the impact of 27 phenotypic traits on the first and second principal components, as well as the contribution rates of the first and second principal components.

After measuring and statistically analyzing 27 quantitative and qualitative traits, we identified eight components with eigenvalues higher than one, which serve as the principal components of the 27 phenotypic traits. These eight principal components cumulatively account for 87.75% of the information in the original traits, with the first and second principal components alone contributing a significant 44.70% (Figure 5). The first principal component is most heavily weighted by the quantity of flowers, closely followed by the height of the scape, indicating that the growth of the scape greatly influences the classification of *Cymbidium* species. In the second principal component, the length of the labellum and petal, and the width of the sepal have substantial weight coefficients, all of which are dimensions of flower size, highlighting the significant role that flower size plays in the classification of *Cymbidium* species. The principal component analysis reveals that these eight components encapsulate the floral traits, encompassing aspects such as the number of flowers, the height of the scape, petals, sepals, labellum, etc. Therefore, the traits of floral phenotype can be used as the main traits for the classification and identification of *Cymbidium* species, while the traits of leaf phenotype and pseudobulb characteristics can serve as supplementary traits for their classification and identification. By describing and analyzing these phenotypic traits as the basis for the classification of *Cymbidium* species, species groups with similar adaptability can be identified, which reflects the genetic and morphological diversity of the species and thus better understands the diversity and evolutionary relationships of *Cymbidium* species.

### 2.5. Cluster Analysis of Phenotypic Traits

**Figure 6:**
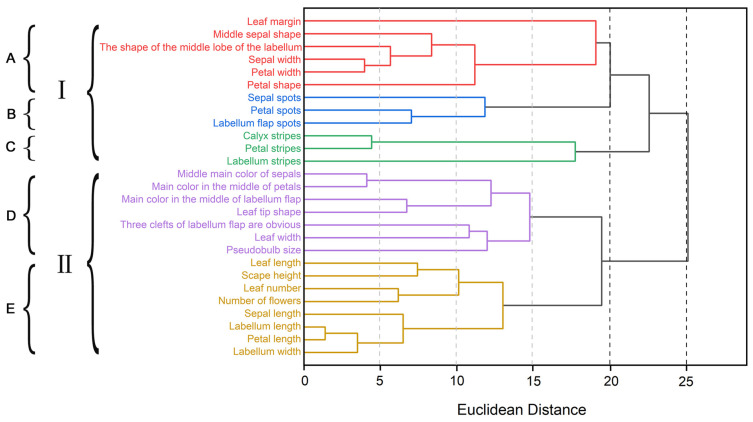
The R-type cluster analysis of 27 phenotypic traits in *Cymbidium* species. When the Euclidean distance was 25 and 20, the 27 phenotypic traits were divided into two groups (I–II) and five groups (A–E).

In order to validate the rationality of trait selection, we conducted in-depth discussions on the correlation between 27 phenotypic traits using R-type clustering analysis. According to the clustering spectrum diagram, when the Euclidean distance was set to 20, we divided the 27 phenotypic traits into 5 groups (Figure 6). Among them, groups A, D, and E contain a relatively large number of phenotypic traits. The five traits of sepal width, petal width, labellum lobe shape, sepal shape, and petal shape in Group A are highly correlated, reflecting the characteristics of flower shape and size. The main color in the middle of the sepals is highly correlated with the main color in the middle of the petals, and the main color in the middle of the labellum is highly correlated with leaf tip traits in Group D. There is also a close correlation between the obvious trichotomy of the labellum, the size of the pseudobulb, and the width of the leaf, and *C*. *elegans* and *C. tracyanum* can be distinguished from other varieties because of the large pseudobulb and the obvious trichotomy of the labellum. The labellum length, labellum width, petal length, and the sepal length and sepal height of Group E are highly correlated, while the number of leaves, number of flowers, leaf length, and scape height are correlated, reflecting the size of flowers and the abundance of plants. This indicates that among the 27 phenotypic traits of *Cymbidium* species, floral characteristics exhibit higher discriminatory power in distinguishing closely related species within the same genus, thus serving as a pivotal factor for classifying the phylogenetic relationships of *Cymbidium* species using phenotypic traits.

**Figure 7:**
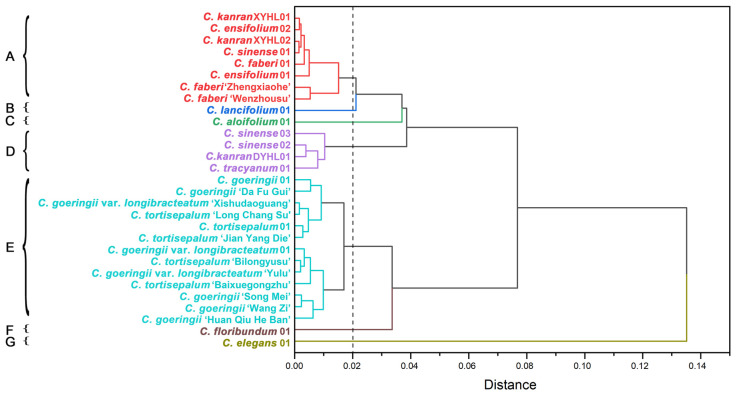
Q-type cluster analysis based on morphological characters depicting the relationship of *Cymbidium* species. All *Cymbidium* species were classified into seven primary clusters: A–G.

Based on the analysis of phenotypic traits, we performed dimensionality reduction on 27 phenotypic traits and ultimately simplified them into 21 phenotypic traits, which were subjected to Q-type clustering analysis. The results revealed significant segregation among various *Cymbidium* species, which were divided into seven clusters (Figure 7). *C. faberi*, *C. kanran*, and *C. ensifolium* were predominantly closely related together, characterized by a longer labellum, petals, and a higher number of flowers in the phenotype. At this level, they have a high degree of similarity and are suitable for clustering together. Because of the high similarity of petal stripes and spots and labellum stripes and spots, *C. sinense* and *C. tracyanum* are gathered in one branch. *C. tortisepalum*, *C. goeringii* var. *longibracteatum*, and *C. goeringii* all exhibit narrow leaves and small pseudobulbs in phenotype, with high similarity at the sublevel, making them suitable for clustering together. The leaf length, number of leaves, scape height, number of flowers, three-lobed labellum, and pseudobulb size of *C. elegans* and *C. floribundum* differ greatly from other terrestrial orchids, while the leaf shapes of *C. lancifolium* and *C. aloifolium* are different from other terrestrial orchids, and their phenotypes are significantly different from those of the A, D, and E clusters, so they are all grouped separately.

### 2.6. The Support of Molecular Characters for Morphological Features

To further discuss the correlation between the combination of phenotype and DNA barcode identification results, we will also conduct an analysis that compares the phenotypic clustering results with the barcode tree construction results, examining the similarities and differences from the perspective of diverse life forms. According to the analysis of phenotypic traits, Figure 7 reveals that *C*. *lancifolium*, *C*. *floribundum*, *C*. *aloifolium*, and *C*. *elegans* each form a distinct branch. The phylogenetic tree (Figure 4) constructed by combining DNA barcodes also demonstrates that *C*. *lancifolium*, *C*. *floribundum*, *C*. *aloifolium*, *C*. *tracyanum*, and *C*. *elegans* were all clustered separately within individual branches. From the R-type cluster analysis of phenotypic traits (Figure 6), we indicated that floral characteristics are the primary morphological features for orchid classification. The floral diagrams of these plant materials among *Cymbidium* in this study showcased a variety of floral traits, including various flower colors, flower spots, and labellum spot variations. Significant differences in the petal patterns were observed between the four epiphytic orchids (*C*. *floribundum*, *C*. *aloifolium*, *C*. *tracyanum*, and *C*. *elegans*) and the terrestrial orchids, as well as among these four epiphytic orchids themselves. When classified using DNA barcodes, these four types of epiphytic orchids were divided into one major category, indicating that the formation of different life forms in *Cymbidium* species is closely related to genes, and *Cymbidium* species with significant morphological differences also share close genetic relationships.

**Figure 8:**
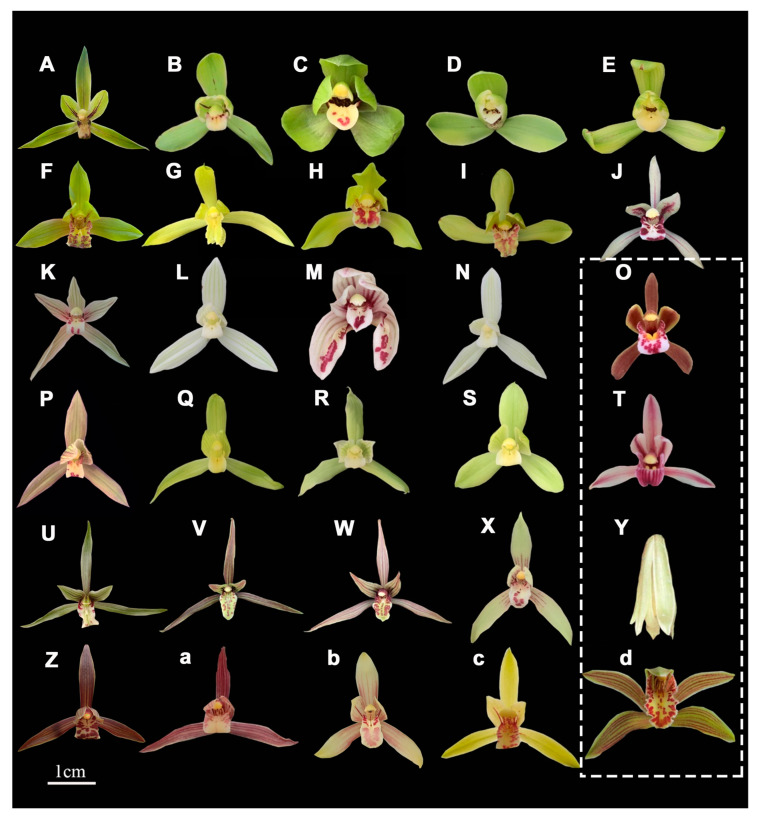
Flower diagram of phenotypic trait test varieties. The white box contains epiphytic orchids, while the rest are terrestrial orchids. (**A**) *Cymbidium goeringii*; (**B**) *Cymbidium goeringii* ‘Song Mei’; (**C**) *Cymbidium goeringii* ‘Huan Qiu He Ban’ (**D**) *Cymbidium goeringii* ‘Da Fu Gui’; (**E**) *Cymbidium goeringii* ‘Wang Zi’; (**F**) *Cymbidium faberi*; (**G**) *Cymbidium faberi* ‘Wenzhousu’; (**H**) *Cymbidium faberi* ‘Zhengxiaohe’; (**I**) *Cymbidium faberi* ‘Dayipin’; (**J**) *Cymbidium lancifolium*; (**K**) *Cymbidium tortisepalum*; (**L**) *Cymbidium tortisepalum* ‘Baixuegongzhu’; (**M**) *Cymbidium tortisepalum* ‘Jian Yang Die’; (**N**) *Cymbidium tortisepalum* ‘Bilongyusu’; (**O**) *Cymbidium floribundum*; (**P**) *Cymbidium goeringii* var. *longibracteatum*; (**Q**) *Cymbidium goeringii* var. *longibracteatum* ‘Xishudaoguang’; (**R**) *Cymbidium tortisepalum* ‘Long Chang Su’; (**S**) *Cymbidium goeringii* var. *longibracteatum* ‘Yulu’; (**T**) *Cymbidium aloifolium*; (**U**) *Cymbidium kanran* 01; (**V**) *Cymbidium kanran* 02; (**W**) *Cymbidium kanran* 04; (**X**) *Cymbidium sinense* var. haematodes; (**Y**) *Cymbidium elegans*; (**Z**) *Cymbidium sinense* ‘Qihei’; (**a**) *Cymbidium sinense* ‘Yangmingjin’; (**b**) *Cymbidium ensifolium* 01; (**c**) *Cymbidium ensifolium* 02; and (**d**) *Cymbidium tracyanum*.

Admittedly, there is a significant divergence in floral characteristics between epiphytic and terrestrial orchids (Figure 8). However, *C. lancifolium*, a species of terrestrial plants, exhibits closer affinities with four epiphytic orchids based on both morphological traits and molecular markers. Although phylogenetic trees may have maximum branch support at their nodes, they can still display a notable lack of consistency in phylogenetic signals derived from different genes or regions of the genome, and additional data may not resolve these inconsistencies. Nonetheless, there is substantial potential to understand the basis of phenotypic variation in orchids, from DNA molecular markers to entire genomes, thereby gaining insight into evolutionary changes and their significance within populations.

## 3. Discussion

The species of *Cymbidium* exhibit extensive morphological and genetic variability due to variations in morphology, genetics, and ecological habits among different species, which poses challenges for species identification and classification. The reasons for these differences in *Cymbidium* plants may be due to variations in the genetic material of *Cymbidium* germplasm and the influence of diverse environmental conditions [26,27,28,29,30]. To adapt to local ecological environments, different genetic traits are produced, and morphological characteristics may not accurately reflect the complete genetic information. Thus, this study employs the more stable and precise DNA barcoding technique to assist us in the classification and identification of *Cymbidium* species.

### 3.1. The Universality and Applicability of Sequences

DNA barcoding technology is an emerging species identification technique that has gained popularity in recent years due to its rapidity, accuracy, and user-friendliness [31]. An ideal DNA barcode should meet three criteria: standardization, extreme simplification, and scalability. It should enable routine and reliable sequencing across diverse sample sets to obtain easily comparable sequences with minimal intraspecific variations but substantial interspecific variations [32]. In this study, we amplified fragments from both nuclear genes (ITS) and chloroplast genes (*matK*, *rbcL*, *psbA-trnH*, *trnL-F*) for barcode analysis. Our findings indicate that there are some differences in PCR amplification and sequencing success rates among the genotypes and loci investigated, which may be related to primer specificity, PCR settings, and the competency of the DNA template. Similar results were also present in previous studies, demonstrating that these factors have a certain impact on the success rates of PCR amplification and sequencing [33]. Sequence feature analysis revealed that ITS exhibited a higher number of variation sites, which was also supported by previous studies on *Uncaria*, *Rhododendron*, and *Gastrodia elata* [34,35,36]. Additionally, the chloroplast gene fragment *psbA-trnH* showed a greater number of variation sites and exhibited a more pronounced barcoding gap. However, both the sequences of *trnL-F* and *matK* displayed relatively low variability in this study, while the variability of *rbcL* was predominantly located at the end of candidate chloroplast genomes without a clear barcoding gap. Therefore, based on the characteristics of these five single-fragment DNA barcodes, ITS and *psbA-trnH* have the potential to serve as effective DNA barcodes for the classification and identification of *Cymbidium* species in this study. Moreover, the specificity of DNA barcoding can reveal the high degree of morphological and genetic variability in *Cymbidium* species. These findings are similar to some previous studies, indicating that DNA barcode regions can serve as reliable markers for the identification and classification of *Cymbidium* species and their genotypes [33,37]. To further validate these results and assess the effectiveness of these barcodes across a broader taxonomic range, additional research is needed to discover and explain the potential of DNA barcodes in distinguishing between closely related species.

### 3.2. Assessment of the Discriminating Power of Single and a Combination of Barcodes

The discriminating power of different DNA barcodes for species identification may vary depending on the taxonomic group studied. Sayed et al. [38] assessed the efficacy of DNA barcodes for species identification and found that *matK* and ITS regions have high universality and sequencing success rates, providing 100% species resolution except for the *trnH-psbA* region. However, in this study, although *matK* and ITS had high sequencing success rates, the *psbA-trnH* sequence, which had a low sequencing success rate, achieved the highest identification success rate and has been successfully applied to identify *Dendrobium* and *Dioscorea* [39,40] as well as other plants. Nevertheless, the species identification success rates for the *trnL-F* sequence and *rbcL* sequence were relatively low. Among the five single-fragment DNA barcodes analyzed in this study, the psbA-trnH sequence demonstrated higher specificity and identification success rate, making it more suitable for the classification and identification of *Cymbidium* species. Relying on a single DNA barcode marker may not always provide sufficient variation information for species identification, thereby presenting certain limitations.

Therefore, the utilization of multiple DNA markers for plant identification has been gradually implemented [22], enabling the acquisition of adequate species variation. For instance, the combination of *arpF-atpH* + *pabK-psbL* + *psbA-trnH* as a DNA barcode has been shown to achieve an impressive species identification rate of 98.8% in orchids [41]. When four DNA fragments were employed to identify *Alnus*, it was observed that the resolution of a single fragment was inferior to that of a combination of multiple fragments [42]. Fazekas et al.’s examination of 251 plant individuals demonstrated mere recognition rates of 44% and 45% for *psbK-psbI* and *atpF-atpH*, respectively; however, when *matk* + *psbK-psbI* + *atpF-atpH* were combined as barcodes, the identification success rate climbed to 69% [43]. Our investigation also revealed that the combination of barcode fragments outperformed single fragments in identifying *Cymbidium* species. Among these combinations, ITS + *matK* + *psbA-trnH* exhibited superior resolution with minimal overlap in the "barcoding gap" among other fragment combinations. Similar verification was conducted within the buckwheat genus, where this sequence combination facilitated accurate identification and supported buckwheat’s monophyletic grouping [44]. Furthermore, in identifying medicinal orchids, complementary performance was observed among *matK*, ITS and *psbA-trnH* sequences. The ITS + *psbA-trnH* sequence displayed substantial genetic variability with improved "barcoding gap" performance. Nevertheless, its effectiveness in identifying *Cymbidium* species was somewhat limited, possibly attributable to insufficient intraspecific variation or the presence of unusually large intraspecific distances.

### 3.3. The Phylogeny of the Cymbidium

Incorporating both sequence feature analysis and genetic distance analysis, the phylogenetic tree was constructed based on *psbA-trnH* of the single-fragment barcodes, *matK* + *psbA-trnH* and ITS + *matK* + *psbA-trnH* of the combined barcodes. It was found that the *Cymbidium* species consisted of six major clades, eight major clades, and five major clades, respectively. Based on the analysis of these three phylogenetic trees, the subgenus *Jensoa* does not appear to form a monophyletic group. Among them, the *C. lancifolium* was embedded in the branches of other subgenera in the phylogenetic tree constructed based on ITS + *matK* + *psbA-trnH*, while it formed a separate small branch in the phylogenetic trees constructed based on the *psbA-trnH* barcode and the combined barcode of *matK + psbA-trnH*. Although most species of the subgenus *Jensoa* were closely related together in the phylogenetic trees constructed based on the *psbA-trnH* and *matK* + *psbA-trnH*, some varieties of the same species were closely related together with other *Cymbidium* species, such as some varieties of *Cymbidium goeringii* (Figure 3). The existence of these differences may be due to natural hybridization between species [45,46,47,48], which led to genetic variation, and may also be due to the lack of plastid information characteristics [22,49], resulting in inconsistent classification results. Which was quite different from the phylogenetic tree by ITS + *matK* + *psbA-trnH*, all samples and varieties of the same species were not separated by the embedding of other species, indicating that the multi-fragment combined barcode enhanced the sequence information characteristics and was more conducive to the classification of the *Cymbidium* species to a certain extent.

During the evolution of orchids, unique characteristics such as deceptive pollination and dust-like, wind-dispersed seeds often lead to gene flow between populations. Deceptive pollination, which involves orchids mimicking sex pheromones and the appearance of specific female insects to sexually lure common male insects as pollinators, allows some orchid species to share pollinators [50]. Therefore, most orchids that use the same pollination strategy and grow in the same habitat share a greater number of insect species than expected. These reproductive strategies make them highly susceptible to interspecific hybridization in nature, resulting in significant genetic heterogeneity between orchid populations [51]. In this study, the analysis of the phylogenetic tree constructed based on the combined barcode revealed that a sample of the epiphytic orchid (*C. floribundum*) was closely related to terrestrial orchids such as *C. ensifolium* in a large clade. It is speculated that this sample of *C. floribundum* may have undergone interspecific hybridization with other *Cymbidium* species. *C. floribundum* primarily relies on insect pollination, and bees are one of the main pollinator groups [52]. Since *C. floribundum* produces a large number of flowers when blooming, it attracts a significant amount of bees as potential pollinators. Given the low specificity of insect pollination, it is likely that during the long cultivation history of *C. floribundum*, cross-pollination with other *Cymbidium* species occurred, leading to genetic changes and variations in the offspring. Additionally, the samples we collected have a relatively longer life history, which may have enabled gene flow between different species via insect pollination in the natural environment. This can lead to genetic variations in *Cymbidium* species without morphological changes, thereby producing classification results that differ from the traditional categorization of *Cymbidium* species.

### 3.4. Identification and Classification of Cymbidium Species Through the Combination of Morphological Analysis and DNA Barcoding Technology

The traditional morphological analysis [53,54] for classifying orchids has certain limitations and deficiencies, as plant characteristics are finite and varying interpretations of phenotypic traits among individuals can result in inconsistent results. In *Cymbidium* genera, phylogenetic trees based on morphology struggle to achieve precise positioning due to rapid dispersal and hybridization, which can induce swift alterations in morphological traits, thus complicating phylogenetic analyses based on morphology [55]. The integration of DNA barcoding technology with morphological analysis offers a more comprehensive basis for classification, from external characteristics to genetic insights. Previous research on phenotypic traits has found that the species of *C. sinense*, *C. ensifolium*, *C. kanran*, and *C. faberi* were closely related, as were the species of *C. goeringii*, *C. goeringii* var. *longibracteatum*, and *C. tortisepalum* [56]. This supports the results of the morphological analysis in this study. Another study, which utilized both morphological analysis and molecular markers, found that *C. kanran* and *C. sinense* cluster in a group, while all species of *C. goeringii*, *C. goeringii* var. *longibracteatum*, and *C. tortisepalum* cluster together, and *C. faberi* forms a separate branch [56]. Although these research findings [56,57,58] have many similarities with this study, they only explore the classification relationships of terrestrial orchids within the *Cymbidium* species. However, our study also investigates the taxonomic status and relationships of epiphytic orchids within the *Cymbidium* species, which indicate that the genetic relationships among epiphytic orchids are different from those of terrestrial orchids and that species with the same life form have closer phylogenetic relationships. Furthermore, the difference is that a species of *C. floribundum* was closely related together with two species of *C. ensifolium* on a small branch. These discrepancies between these two research methods have also been observed in the identification of *Amorphophallus* and *Rhododendron* [34,59]. However, discrepancies are noted in the classification relationships among the *Cymbidium* species known as *C. kanran*, *C. floribundum*, and *C. lancifolium*; the taxonomic status of these three species of *Cymbidium* requires further investigation.

It is noteworthy that *C. lancifolium*, which has some phenotypic differences from the typical terrestrial orchids in the *Cymbidium* species, clusters with the majority of terrestrial species within the *Cymbidium* in some research reports, indicating a closer phylogenetic relationship with terrestrial orchids [60,61]. However, other studies [62] on the classification of *Cymbidium* species show that *C. lancifolium* forms a separate cluster, which is consistent with the phenotypic trait clustering results of this study. Although this differs from the phylogenetic tree analysis results within this study, which indicated that *C. lancifolium* grouped with epiphytic orchid species (such as *C. aloifolium*, *C. tracyanum*, *C. elegans*, etc.) in Clade IV (Figure 4), it still indicates a closer phylogenetic relationship with epiphytic orchids. We posit that *C. lancifolium* has a potential that is more inclined towards epiphytism, making it closer to epiphytic orchids. It is speculated that this may be related to the origins and evolution of terrestrial orchids and epiphytic orchids. The origin and evolution of epiphytic orchids are similar to the various origins of ferns, Bromeliaceae, and eudicots [63,64,65,66], suggesting that the transformation of epiphytism may rely on certain morphological or genetic prerequisites that are common among members of large taxonomic groups. Given the discrepancies among different research findings [22], there is a need for more accurate molecular identification methods to precisely determine its classification and life form.

Our research findings highlight the practical application of the barcode method in accurately determining the geographical origins of orchid specimens. This capability is of great significance for managing the legal trade in medicinal and ornamental orchids, ensuring that such trade does not adversely affect threatened populations. In addition, by enabling law enforcement agencies to assign geographical origins to unknown specimens extracted in illegal trade, barcode technology has great potential in combating illegal trade and conserving biodiversity. However, we also recognize that, in order to realize its full potential, barcoding should be used in conjunction with other verification methods and implemented within a broader policy and regulatory framework.

### 3.5. Cymbidium Species Diversity

In recent years, research on molecular markers has mainly focused on selecting the most suitable barcode for specific families and genera [18,20]. The use of certain single-gene fragments or combinations currently cannot distinguish all higher plants and is limited to a narrow range, such as family, genus, and species levels [67,68]. Even within a specific range, the results may sometimes be erroneous or contradictory to traditional morphological classification. Moreover, the majority of studies [69,70,71,72,73] on orchid morphology or genetic evolution primarily focus on epiphytic orchids within the entire orchid family or subfamily, with limited analysis conducted on species diversity within *Cymbidium* species and particularly the evolutionary disparities among *Cymbidium* species from different regions.

In this study, the phylogenetic tree constructed using single-fragment barcodes was not as effective in species identification and classification as the phylogenetic tree constructed using combined barcodes. Yang et al. also found that, with the increase in sequences, the phylogenetic resolution and node support values significantly improved, and phylogenetic analyses based on the complete chloroplast genome could overcome the limitations of insufficient DNA sequence sampling [17]. Previous studies, although providing better identification and classification of *Cymbidium* species, did not explore the possible reasons for such classification results. A recent study using the chloroplast genome of *Cymbidium* species speculated that the ancestral life form of *Cymbidium* species was epiphytic and discovered that the northernmost species of the genus had undergone three transitions from epiphytic to terrestrial habits, which seemed to be related to adaptation to the colder northern environment [61]. In this study, a sample of the epiphytic Orchis (*C. floribundum*) collected in Hunan, China, was closely related to some terrestrial Orchis also collected in Hunan in the phylogenetic tree, which is likely due to genetic variation in internal structure for adaptation to local climate change. Climate change is widely recognized as a significant driver of species diversity, and its impact on other plant lineages has been extensively studied [74,75,76]. In particular, monsoon climates have been shown to shape the evolutionary trajectories of many plant species [77]. Climate change and geographical distribution together influence the life-form changes of *Cymbidium* species and affect their diversity by altering their living environments [61]. In this study, species collected in southern China were predominantly epiphytic, while those collected further north were terrestrial. The phylogenetic tree classification results indicated that species with the same life form had closer phylogenetic relationships. This indicates that the phylogenetic tree constructed using DNA barcoding technology can, to some extent, analyze the geographical origins of species.

The phylogenetic perspective is greatly helpful for understanding the evolutionary development of *Cymbidium* species, but the process of morphological evolution in *Cymbidium* species is highly complex. Amidst the increasing habitat changes caused by humans, many types of orchids are still being discovered and statistically described, which increases the chance of documenting diversity. Therefore, it is imperative to employ genome sequences of *Cymbidium* species in future research to explicate their adaptability to various environments and investigate the evolution of diverse phenotypes and forms. Comparative genomics serves as a potent tool for studying evolution and morphology, while genome assembly will provide invaluable resources for identifying genetic variations associated with ecological traits in *Cymbidium* species and facilitating genomics-assisted breeding.

## 4. Materials and Method

### 4.1. Plant Materials

Germplasm resources of the *Cymbidium* species were collected in regions including Hunan, Yunnan, Guangdong, and Fujian, compiling a total of 48 individuals from 30 *Cymbidium* species resources, including species, varieties, and cultivars [7,78]. This study selected 30 species of *Cymbidium*. to serve as taxonomic units for the classification analysis of phenotypic traits. The specimen details are presented in Table S5. To enhance the controllability of the data, 48 individuals from 30 species were used to assess the success rate of PCR amplification and sequencing. Species information is detailed in Table S6. Leaf samples from *Cymbidium* species were collected in batches, and DNA was extracted to evaluate the success rate of PCR amplification and sequencing. All experimental materials are maintained and managed in the flower garden of Hunan Agricultural University, with the requirement that the plants tested be healthy and free from diseases.

### 4.2. DNA Extraction, Amplification, and Sequencing

The total DNA of *Cymbidium* plants was extracted using the Polysaccharide Polyphenol Plant Genomic DNA Extraction Kit (DP360) from Tiangen Biochemical Technology Co., Ltd. (Beijing, China). The specific steps are described in the instructions. The extracted total DNA was subjected to concentration determination and stored at −20 °C for later use. Through a literature review and repeated amplification tests, the universal primer sequences of ITS, *matK*, *rbcL*, *psbA-trnH*, and *trnL-F* were ultimately selected. The detailed information of the primers and reaction procedures is presented in Table 6, with the primers synthesized by Shanghai Sangon Bioengineering Co., Ltd. (Shanghai, China). PCR amplification products with bright bands, high specificity, and correct fragment size were chosen and submitted to Shanghai Sangon Bioengineering Co., Ltd. for bidirectional sequencing. Because of the varying barcode characteristics, the number of orchid genus sequences obtained from sequencing is unequal (Table S7).

### 4.3. Data Analysis

The sequence peak image files obtained from sequencing were corrected and concatenated using the Seqman 7.1 software, and the BLAST (Basic Local Alignment Search Tool) online tool (https://blast.ncbi.nlm.nih.gov/Blast.cgi, accessed on 7 January 2025) was used for alignment verification. With MEGA 11.0 software and a genetic distance model based on the Kimura two-parameter (K2-P) method [85], we calculated the intra- and inter-species genetic distances for each barcode of *Cymbidium* species and compared the sequence differences between intra- and interspecific variations [86,87,88]. An area chart of the frequency distribution of genetic distance was plotted using Origin 2022, which facilitates a clear observation of whether there is a barcoding gap in each DNA barcode sequence within *Cymbidium* species [87].

Phylogenetic trees provide an intuitive assessment of the species identification ability of DNA barcodes and are among the evaluation criteria for DNA barcodes [89]. In this study, we employed the neighbor-joining method in the MEGA 11.0 software to construct a phylogenetic tree. The K2-P genetic distance model was selected, with the bootstrap test set to 1000 replications [90]. The bootstrap values support rate needs to be greater than or equal to 50% for accurate and reliable identification results.

Phenotypic trait variability and correlation analyses were performed using Excel 2022 and Spss 27.0. A principal component analysis of 27 phenotypic traits was conducted using Origin 2022. Additionally, R-type cluster analysis was employed to perform a correlation analysis on the 27 phenotypic traits, and Q-type cluster analysis based on phenotypic traits was used to conduct a qualitative analysis of the relationships among the 30 species of *Cymbidium*.

### 4.4. Selection and Determination Methods of Phenotypic Traits

In accordance with the testing guidelines for the specificity, consistency, and stability of orchid species issued by the Ministry of Agriculture of the People’s Republic of China (TG/164/3), 27 representative phenotypic traits such as leaf length, sepal length, petal traits, and middle sepal traits were selected, including 11 quantitative traits (Table S8) and 16 qualitative traits (Table S9). During the full-bloom stage of each species, the quantitative traits were measured using tapes and vernier calipers as detailed in Table S8, while qualitative traits were coded and assigned as shown in Table S9. The traits related to color were obtained with reference to the Royal Horticultural Society’s standard color card. Ultimately, the average of the actual measured values was calculated, and both the quantitative traits and the assigned qualitative traits were documented and analyzed.

## 5. Conclusions

Based on the similarities between cluster results and phylogenetic tree results, we verified and selected the combination sequence ITS + *matK* + *psbA-trnH* as a high-quality DNA barcode for *Cymbidium* species. Furthermore, by constructing a phylogenetic tree with combined barcodes, we observed that epiphytic orchid species clustered together on a single clade, and species with similar life forms were closely related. Our study also revealed that phenotypic analysis successfully classified the germplasm resources of *Cymbidium* into seven groups. Floral phenotypic traits were confirmed as the primary characteristics for differentiating both interspecific and intraspecific variations within *Cymbidium* species. The aforementioned findings not only elucidate the application of DNA barcoding techniques for *Cymbidium* species classification but also enhance our comprehension of the taxonomy and geographic distribution of *Cymbidium* species. Simultaneously, they foster deeper contemplation of the taxonomic relationships among diverse life forms of *Cymbidium* species while offering novel ideas and insights for future research on genetic diversity and adaptive evolution within different life forms of *Cymbidium* species.

## Figures and Tables

**Table 1:** Amplification and sequencing of DNA barcoding.

DNA Barcodes	Number of Samples	Number of Successful Amplifications	Amplification Success Rate	Number of Successful Sequences	Sequencing Success Rate
ITS	48	48	100%	46	95.8%
*matK*	48	48	100%	48	100%
*rbcL*	48	48	100%	48	100%
*psbA-trnH*	48	48	100%	46	95.8%
*trnL-F*	48	48	100%	46	95.8%

**Table 2:** The sequence features of the DNA barcoding.

Sequence Information	ITS	*matK*	*rbcL*	*trnL-F*	*psbA-trnH*
Comparison length (bp)	885	861	645	775	865
GC content (%)	63.6%	31.1%	41.1%	34.62%	33.36%
Conserved site	672 (75.93%)	784 (91.06%)	627 (97.21%)	721 (93.03%)	732 (84.62%)
Total variation sites	165 (18.64%)	73 (8.48%)	17 (2.64%)	23 (2.97%)	96 (11.10%)
Parsimony-informative site	49	40	10	11	74

**Table 3:** DNA combination barcode sequence characteristics.

Sequence Information	ITS + *matK*	*matK* + *psbA-trnH*	ITS + *psbA-trnH*	ITS + *psbA-trnH* + *matK*
Comparison length (bp)	1716	1726	1720	2581
GC content (%)	48.1%	32.5%	50.3%	43.6%
Conserved site	1399 (81.53%)	1516 (87.83%)	1367 (79.48%)	2162 (83.77%)
Total variation sites	303 (17.66%)	169 (9.79%)	306 (17.79%)	368 (14.26%)
Parsimony-informative site	124	114	154	188
Single polymorphic loci	179	54	151	179

**Table 4:** Comparison of genetic distance differences of single-segment DNA barcodes.

DNA Barcodes	Intraspecific Genetic Distance			Interspecific Genetic Distance		
	Minimum	Maximum	Mean	Minimum	Maximum	Mean
ITS	0	0.09657	0.01759	0	0.14136	0.02890
*matk*	0	0.02262	0.00335	0	0.03115	0.01208
*rbcL*	0	0.00782	0.00088	0	0.01731	0.00424
*trnL-F*	0	0.00980	0.00168	0	0.02004	0.00434
*psbA-trnH*	0	0.02128	0.00513	0	0.04525	0.01588

**Table 5:** Comparison of genetic distance differences of DNA combination barcodes.

DNA Barcodes	Intraspecific Genetic Distance			Interspecific Genetic Distance		
	Minimum	Maximum	Mean	Minimum	Maximum	Mean
ITS + *matK*	0	0.04979	0.01041	0	0.07025	0.02025
ITS + *psbA-trnH*	0	0.04855	0.01147	0	0.09987	0.02258
*matK* + *psbA-trnH*	0	0.02387	0.00409	0	0.04290	0.01366
ITS + *matK* + *psbA-trnH*	0	0.03395	0.00824	0	0.06201	0.01841

**Table 6:** PCR primers and reaction procedures of five DNA barcodes.

Barcodes	Primer Name	Primer Sequence (5′–3′)	Reaction Procedure	References
ITS	17SE	ACGAATTCATGGTCCGGTGAAGTGTTCG	95 °C 3 min, 35 cycle (95 °C 15 s, 62 °C 15 s, 72 °C 15 s), 72 °C 5 min	Sun et al., 1994 [79]
	26SE	TAGAATTCCCCGGTTCGCTCGCCGTTAC		Sun et al., 1994
*matK*	390F	CGATCTATTCATTCAATATTTC	95 °C 3 min, 35 cycle (95 °C 15 s, 46.5 °C 15 s, 72 °C 15 s), 72 °C 5 min	Cuenoud et al., 2002 [80]
	1326R	TCTAGCACACGAAAGTCGAAGT		Cuenoud et al., 2002
*psbA-trnH*	psbA	GTTATGCATGAACGTAATGCTC	95 °C 3 min, 35 cycle (95 °C 15 s, 55 °C 15 s, 72 °C 15 s), 72 °C 5 min	Sang et al., 1997 [81]
	trnH2	CGCGCATGGTGGATTCACAATCC		Tate, 2002 [82]
*rbcL*	1F	ATGTCACCACAAACAGAAAC	95 °C 3 min, 35 cycle (95 °C 15 s, 56 °C 15 s, 72 °C 15 s), 72 °C 5 min	Goldman et al., 2001 [83]
	724R	TGCCATGTACCYGCAGTTGC		Goldman et al., 2001
*trnL-F*	c	CGAAATCGGTAGACGCTACG	95 °C 3 min, 35 cycle (95 °C 15 s, 53 °C 15 s, 72 °C 15 s), 72 °C 5 min	Taberlet et al., 1991 [84]
	f	ATTTGAACTGGTGACACGAG		Taberlet et al., 1991

## Data Availability

The raw data supporting the conclusions of this article will be made available by the authors upon request.

## Supplementary Materials

The following supporting information can be downloaded at https://www.mdpi.com/article/10.3390/plants14040619/s1. Table S1: *Cymbidium* species resolution is based on the method of genetic distance with “best match”, “best close match”, and a phylogenetic tree of five barcodes and their combination. Table S2: Variation of 11 quantitative traits in *Cymbidium* species. Table S3: Correlation analysis of 11 quantitative traits of *Cymbidium* species. Table S4: Frequency distribution of 16 quality traits of *Cymbidium* species. Table S5: *Cymbidium* species samples and their information used to determine phenotypic traits in this study. All plant samples were maintained and managed in the flower base of Hunan Agricultural University. Table S6: 48 plant samples of *Cymbidium* and information used for DNA extraction in this study. All plant samples were maintained and managed in the flower base of Hunan Agricultural University. Table S7: GenBank accession numbers of the five loci sequences for *Cymbidium* species examined in this study. Table S8: Quantitative traits and test methods of *Cymbidium* spp. Table S9: Quality traits and its assignment of *Cymbidium* spp.
