# Supplementary material for: From Phenotypes to Genotypes: Enhancing the Identification of Cymbidium Species with DNA Barcoding
Source: Plants (Basel). 2025 Feb 18;14(4):619. doi: 10.3390/plants14040619 (PMC11859091; doi:10.3390/plants14040619)
Supplement: Supplementary file 1 [file plants-14-00619-s001.zip › plants-3441988-supplementary.pdf]

Supplementary materials

Table S1. *Cymbidium* species resolution is based on the method of genetic distance with "best match", "best close match" and a phylogenetic tree of five barcodes and their combination.

Barcodes	All Species Barcodes			Best Match			Best Close Match		
	Correct	Ambiguous	Incorrect	Correct	Ambiguous	Incorrect	Correct	Ambiguous	Incorrect
ITS	10.63%	89.36%	0.00%	38.29%	21.27%	40.42%	38.29%	21.27%	40.42%
matK	43.75%	52.08%	4.16%	41.66%	52.08%	6.25%	41.66%	52.08%	6.25%
rbcL	49.58%	46.25%	0.00%	27.08%	64.58%	4.16%	27.08%	64.58%	4.16%
trnL-F	27.27%	61.36%	9.09%	11.36%	77.27%	11.36%	11.36%	77.27%	9.09%
psbA-trnH	45.65%	47.82%	6.52%	41.30%	54.34%	4.34%	41.30%	54.34%	4.34%
ITS+ *matK*	12.76%	85.10%	2.12%	46.80%	4.25%	48.93%	46.80%	4.25%	48.93%
ITS+ *psbA-trnH*	17.39%	82.60%	0.00%	43.47%	19.56%	36.95%	43.47%	19.56%	36.95%
matK+ psbA-trnH	21.73%	73.91%	4.34%	52.17%	41.30%	6.52%	52.17%	41.30%	6.52%
ITS+ *matK+ psbA-trnH*	22.22%	75.55%	0.00%	55.55%	6.66%	37.77%	55.55%	6.66%	37.77%

Table S2. Variation of 11 quantitative traits in *Cymbidium* specie.

Character	Max	Min	Poor	Average	Variance	Standard deviation	Coefficient of variation
Leaf length (cm)	69.2	21.2	47.8	44.83	160.85	12.68	28.20%
Leaf width (cm)	5.3	0.5	4.8	1.37	0.95	0.98	71.50%
Leaf number (pieces)	14.3	3	11	5.98	5.08	2.25	37.60%
Sepal length (cm)	6.11	1.8	4.31	3.21	0.91	0.96	29.90%
Sepal width (cm)	2.1	0.45	1.65	0.89	0.14	0.37	41.60%
Labellum length (cm)	6.2	1.01	5.19	2.28	0.81	0.9	39.50%
Labellum width (cm)	2.4	0.64	1.76	1.03	0.12	0.34	33.00%
Petal length (cm)	7.3	1.69	5.61	2.6	1.05	1.02	39.20%
Petal width (cm)	1.4	0.6	0.8	0.86	0.06	0.24	27.90%
Scape height (cm)	63.3	6.5	56.8	25.35	243.11	15.59	61.50%
Number of flowers (number)	41.5	1	40.5	5.88	57.51	7.58	128.90%

Table S3. Correlation analysis of 11 quantitative traits of *Cymbidium* species.

Indicators	Leaf length	Leaf width	Leaf number	Sepal length	Sepal width	Labellum length	Labellum width	Petal length	Petal width	Scape height	Number of flowers
Leaf length	1	-0.05	0.543**	0.442*	-0.072	0.495**	0.364	0.456*	-0.174	0.667**	0.493**
Leaf width		1	-0.075	-0.097	-0.223	0.187	0.321	0.17	-0.357	0.291	0.277
Leaf number			1	0.202	0.296	0.454*	0.520**	0.383*	0.057	0.490**	0.723**
Sepal length				1	0.323**	0.725**	0.568**	0.832**	0.202	0.399*	0.028
Sepal width					1	0.493**	0.510**	0.513**	0.823**	-0.068	-0.162
Labellum length						1	0.853**	0.9338**	0.127	0.661**	0.321
Labellum width							1	0.833**	0.135	0.620**	0.497*
Petal length								1	0.185	0.580**	0.233
Petal width									1	-0.405*	-0.440*
Scape height										1	0.659**
Number of flowers											1

Note: * indicates significant correlation, ** indicates extremely significant correlation.

Table S4. Frequency distribution of 16 quality traits of *Cymbidium* species.

Character	Distribution								Shannon-Weiner index
	1	2	3	4	5	6	7	8	
Middle main color of sepals	21.90%	46.20%	15.20%			6.70%	6.60%	3.30%	2.2
Calyx stripes	19.90%	80.10%							0.66
Sepal spots	73.30%	26.70%							0.8
Middle sepal shape	13.30%	10%	53.30%	13.30%	10%				1.69
Main color in the middle of petals	23.30%	50%	16.70%			3.40%	6.70%		1.92
Petal shape		20%	60%	16.70%	3.30%				1.46
Petal spots	43.30%	56.70%							0.99
Main color in the middle of labellum flap	40%	13.30%	43.30%				3.30%		1.55
Petal stripes	13.30%	86.70%							0.48
Labellum flap spots	26.70%	73.30%							0.85
Labellum stripes	70%	30%							0.85
The shape of the middle lobe of the labellum	20%	43.30%		30%	6.70%				1.79
Three clefts of labellum flap are obvious	66.70%	23.30%	10%						1.23
Pseudobulb size	13.30%	63.30%	10%	13.30%					1.31
Leaf margin	23.30%	63.30%	13.30%						1.23
Leaf tip shape	96.70%		3.30%						0.22

Table S5. *Cymbidium* species samples and their information used to determine phenotypic traits in this study. All plant samples were maintained and managed in the flower base of Hunan Agricultural University.

**NO.**	**Species name**	**Collection site**
CL01	*Cymbidium goeringii* Rchb.f.	Yueyang, Hunan, China
CL 'Song Mei'	*Cymbidium goeringii* 'Song Mei'	Zhangzhou, Fujian, China
CL 'Da Fu Gui'	*Cymbidium goeringii* 'Da Fu Gui'	Zhangzhou, Fujian, China
CL 'Huan Qiu He Ban'	*Cymbidium goeringii* 'Huan Qiu He Ban'	Zhangzhou, Fujian, China
CL 'Wang Zi'	Cymbidium goeringii 'Wang Zi	Zhangzhou, Fujian, China
HL01	*Cymbidium faberi* Rolfe	Zhangjiajie, Hunan, China
HL 'Wenzhousu'	*Cymbidium faberi* 'Wenzhousu'	Zhangzhou, Fujian, China
HL 'Zhengxiaohe'	*Cymbidium faberi* 'Zhengxiaohe'	Zhangzhou, Fujian, China
XYHL01	*Cymbidium kanran* Makino	Zhuzhou, Hunan, China
XYHL02	*Cymbidium kanran* Makino	Yifeng, Jiangxi, China
DYHL01	*Cymbidium kanran* Makino	Xiangxi, Hunan, China
ML01	*Cymbidium sinense* var. *haematodes*	Wengyuan, Guangdong, China
ML02	*Cymbidium sinense* 'Qihei'	Wengyuan, Guangdong, China
ML03	*Cymbidium sinense* 'Yangmingjini'	Wengyuan, Guangdong, China
JL01	*Cymbidium ensifolium* (L.) Sw.	Yueyang, Hunan, China
JL02	*Cymbidium ensifolium* (L.) Sw.	Yueyang, Hunan, China
CJ01	*Cymbidium goeringii* var. *longibracteatum* Y.S.Wu et S.C.Chen	Liuzhou, Guangxi, China
CJ 'Xishudaoguang'	*Cymbidium goeringii* var. *longibracteatum* 'Xishudaoguang'	Zhangzhou, Fujian, China
CJ 'Yulu'	*Cymbidium goeringii* var. *longibracteatum* 'Yulu'	Zhangzhou, Fujian, China
LBL01	*Cymbidium tortisepalum* Fukuy.	Baoshan, Yunnan, China
LBL 'Bilongyusu'	*Cymbidium tortisepalum* 'Bilongyusu'	Baoshan, Yunnan, China
LBL 'Jian Yang Die'	*Cymbidium tortisepalum* 'Jian Yang Die'	Baoshan, Yunnan, China
LBL 'Long Chang Su'	*Cymbidium tortisepalum* 'Long Chang Su'	Zhangzhou, Fujian, China
LBL 'Baixuegongzhu'	*Cymbidium tortisepalum* 'Baixuegongzhu'	Baoshan, Yunnan, China
TEL01	*Cymbidium lancifolim* Hook.	Chenzhou, Hunan, China
DHL01	*Cymbidium floribundum* Lindl.	Baoshan, Yunnan, China
SCL01	*Cymbidium elegans* Lindl.	Baoshan, Yunnan, China
XZHTL01	*Cymbidium tracyanum* Rolfe	Baoshan, Yunnan, China
WBL01	*Cymbidium aloifolium* (L.) Sw.	Baoshan, Yunnan, China

Table S6. 48 plant samples of *Cymbidium* and information used for DNA extraction in this study. All plant samples were maintained and managed in the flower base of Hunan Agricultural University.

NO.	Species name	Collection site
CL01	*C. goeringii* Rchb.f.	Yueyang, Huanan, China
CL02	*C. goeringii* Rchb.f.	Yueyang, Huanan, China
CL03	*C. goeringii* Rchb.f.	Yueyang, Huanan, China
CL 'Song Mei'	*C. goeringii* 'Song Mei'	Zhangzhou, Fujian, China
CL 'Da Fu Gui'	*C. goeringii* 'Da Fu Gui'	Zhangzhou, Fujian, China
CL 'Huan Qiu He Ban'	*C. goeringii* 'Huan Qiu He Ban'	Zhangzhou, Fujian, China
HL01	*C. faberii* Rolfe	Zhangjiajie, Hunan, China
HL02	*C. faberii* Rolfe	Zhangjiajie, Hunan, China
HL03	*C. faberii* Rolfe	Zhangjiajie, Hunan, China
HL 'Dayipin'	*C. faberi* 'Dayipin'	Zhangzhou, Fujian, China
HL 'Wenzhousu'	*C. faberi* 'Wenzhousu'	Zhangzhou, Fujian, China
HL 'Zhengxiaohe'	*C. faberi* 'Zhengxiaohe'	Zhangzhou, Fujian, China
XYHL01	*C. kanran* Makino	Zhuzhou, Hunan, China
XYHL02	*C. kanran* Makino	Yifeng, Jiangxi, China
XYHL03	*C. kanran* Makino	Zhuzhou, Hunan, China
DYHL04	*C. kanran* Makino	Xiangxi, Hunan, China
DYHL05	*C. kanran* Makino	Xiangxi, Hunan, China
DYHL06	*C. kanran* Makino	Xiangxi, Hunan, China
ML01	*C. sinense* var. *haematodes*	Wengyuan, Guangdong, China
ML02	*C. sinense* 'Qihei'	Wengyuan, Guangdong, China
ML03	*C. sinense* 'Qihei'	Wengyuan, Guangdong, China
JL01	*C. ensifolium* (L.) Sw.	Yueyang, Hunan, China
JL02	*C. ensifolium* (L.) Sw.	Yueyang, Hunan, China
JL03	*C. ensifolium* (L.) Sw.	Loudi, Hunan, China
CJ01	*C. goeringii var. longibracteatum* Y.S.Wu et S.C.Chen	Liuzhou, Guangxi, China
CJ02	*C. goeringii var. longibracteatum* Y.S.Wu et S.C.Chen	Liuzhou, Guangxi, China
CJ03	*C. goeringii var. longibracteatum* Y.S.Wu et S.C.Chen	Liuzhou, Guangxi, China
CJ 'Xishudaoguang'	*C. goeringii var. longibracteatum* 'Xishudaoguang'	Zhangzhou, Fujian, China
CJ 'Yulu'	*C. goeringii var. longibracteatum* 'Yulu'	Zhangzhou, Fujian, China
LBL 'Long Chang Su'	*C. tortisepalum* 'Long Chang Su'	Zhangzhou, Fujian, China
LBL01	*C. tortisepalum* Fukuy.	Baoshan, Yunnan, China
LBL 'Bilongyusu'	*C. tortisepalum* 'Bilongyusu'	Baoshan, Yunnan, China
LBL 'Jian Yang Die'	*C. tortisepalum* 'Jian Yang Die'	Baoshan, Yunnan, China
LBL 'Baixuegongzhu'	*C. tortisepalum* 'Baixuegongzhu'	Baoshan, Yunnan, China
TEL01	*C. lancifolium* Hook.	Bijie, Guizhou, China
TEL02	*C. lancifolium* Hook.	Bijie, Guizhou, China
TEL03	*C. lancifolium* Hook.	Bijie, Guizhou, China
DHL01	*C. floribundum* Lindl.	Chenzhou, Hunan, China
DHL02	*C. floribundum* Lindl.	Chenzhou, Hunan, China
DZC01	*C. eburneum* Lindl.	Baoshan, Yunnan, China
DZC02	*C. eburneum* Lindl.	Baoshan, Yunnan, China
SCL01	*C. elegans* Lindl.	Baoshan, Yunnan, China

(Continuation Table S6)

NO.	Species name	Collection site
SCL02	*C. elegans* Lindl.	Baoshan, Yunnan, China
SCL03	*C. elegans* Lindl.	Baoshan, Yunnan, China
XZHTL01	*C. tracyanum* Rolfe	Baoshan, Yunnan, China
XZHTL02	*C. tracyanum* Rolfe	Baoshan, Yunnan, China
WBL01	*C. aloifolium* (L.) Sw.	Baoshan, Yunnan, China
WBL02	*C. aloifolium* (L.) Sw.	Baoshan, Yunnan, China

Table S7. GenBank accession numbers of the five loci sequences for *Cymbidium* species examined in this study.

NO.	*ITS*	*matK*	*psbA-trnH*	*rbcL*	*trnL-F*
CL01	PQ809727	PQ815767	PQ815672	PQ815720	PQ815625
CL02	PQ809728	PQ815768	PQ815671	PQ815719	PQ815624
CL03	PQ809729	PQ815769	PQ815673	PQ815721	PQ815626
CJ01	PQ809730	PQ815770	PQ815676	PQ815725	PQ815630
CJ02	/	PQ815771	PQ815675	PQ815726	PQ815631
CJ03	PQ809731	PQ815772	PQ815674	PQ815724	PQ815629
DYHL04	PQ809732	PQ815773	PQ815677	PQ815727	PQ815632
DYHL05	PQ809733	PQ815774	PQ815678	PQ815728	PQ815633
DYHL06	PQ809734	PQ815775	PQ815679	PQ815729	PQ815634
DZC01	/	PQ815776	PQ815680	PQ815731	PQ815636
DZC02	PQ809735	PQ815777	PQ815681	PQ815732	PQ815637
DHL01	PQ809736	PQ815778	PQ815682	PQ815733	PQ815638
DHL02	PQ809737	PQ815779	PQ815683	PQ815734	PQ815639
XZHTL01	PQ809738	PQ815780	PQ815684	PQ815737	PQ815642
XZHTL02	PQ809739	PQ815781	PQ815685	PQ815738	PQ815643
HL02	PQ809740	PQ815782	/	PQ815740	PQ815645
HL03	PQ809741	PQ815783	PQ815686	PQ815741	PQ815646
HL01	PQ809742	PQ815784	PQ815687	PQ815742	PQ815647
JL01	PQ809743	PQ815785	PQ815688	PQ815743	PQ815648
JL03	PQ809744	PQ815786	PQ815689	PQ815744	PQ815649
JL02	PQ809745	PQ815787	PQ815690	PQ815745	PQ815650
LBL01	PQ809746	PQ815788	PQ815691	PQ815746	/
ML02	PQ809747	PQ815789	PQ815692	PQ815748	PQ815652
ML03	PQ809748	PQ815790	PQ815693	PQ815749	PQ815653
ML01	PQ809749	PQ815791	PQ815694	PQ815750	/
SCL01	PQ809750	PQ815792	PQ815695	PQ815752	PQ815656
SCL02	PQ809751	PQ815793	PQ815696	PQ815753	PQ815657
SCL03	PQ809752	PQ815794	PQ815697	PQ815754	PQ815658
TEL01	PQ809753	PQ815795	PQ815698	PQ815756	PQ815660
TEL02	PQ809754	PQ815796	PQ815699	PQ815757	PQ815661
TEL03	PQ809755	PQ815797	PQ815700	PQ815758	PQ815662
WBL01	PQ809756	PQ815798	PQ815701	PQ815760	PQ815664
WBL02	PQ809757	PQ815799	PQ815702	PQ815761	PQ815665
XYHL01	PQ809758	PQ815800	PQ815703	PQ815762	PQ815666
XYHL03	PQ809759	PQ815801	PQ815704	PQ815763	PQ815667
XYHL02	PQ809760	PQ815802	PQ815705	PQ815764	PQ815668
LBL 'Baixuegongzhu'	PQ809761	PQ815803	PQ815706	PQ815722	PQ815627
LBL 'Bilongyusu'	PQ809762	PQ815804	PQ815707	PQ815723	PQ815628
LBL 'Jian Yang Die'	PQ809763	PQ815805	PQ815708	PQ815730	PQ815635
CL 'Da Fu Gui'	PQ809764	PQ815806	PQ815709	PQ815735	PQ815640
CJ 'Xishudaoguang'	PQ809765	PQ815807	PQ815710	PQ815736	PQ815641
CL 'Huan Qiu He Ban'	PQ809766	PQ815808	PQ815711	PQ815739	PQ815644
LBL 'Long Chang Su'	PQ809767	PQ815809	/	PQ815747	PQ815651
HL 'Dayipin'	PQ809768	PQ815810	PQ815712	PQ815751	PQ815655
CL 'Song Mei'	PQ809769	PQ815811	PQ815713	PQ815755	PQ815659

(Continuation Table S7)

NO.	*ITS*	*matK*	*psbA-trnH*	*rbcL*	*trnL-F*
CJ 'Yulu'	PQ809770	PQ815813	PQ815715	PQ815765	PQ815669
HL 'Zhengxiaohe'	PQ809771	PQ815814	PQ815716	PQ815766	PQ815670
HL 'Wenzhousu'	PQ809772	PQ815812	PQ815714	PQ815759	PQ815663

Table S8. Quantitative traits and test methods of *Cymbidium* spp.

NO.	Character	Test methods
1	Leaf length	Measuring the length of the longest leaf in a single plant
2	Leaf width	The widest part of the longest leaf of a single plant
3	Number of leaves	Number of leaves in a single plant
4	Sepal length	Mean of sepal lengths on both sides
5	Sepal width	Mean value of sepal width on both sides
6	Labellum length	Distance from the base of the labrum to the top of the labrum when the labrum is unfolded
7	Labellum width	The widest part of the labellum when they are spread
8	Petal length	Average of the lengths of the two petals
9	Petal width	Average of the width of the two petals
10	Length of scape	Length from the base of the scape to the top flower
11	Number of flowers in the scape	Number of flowers in a single scape

Table S9. Quality traits and its assignment of *Cymbidium* spp.

NO.	Character	Trait expressions and assignments
1	Middle sepal primary color	(1) White (2) Green (3) Yellow (4) Orange (5) Pink (6) Red (7) Purple
2	Sepal stripes	(1) None (2) Yes
3	Sepal spot	(1) None (2) Yes
4	Middle sepal shape	(1) Lanceolate (2) Linear (3) Oblong (4) Elliptic (5) Obovate
5	Petal Color	(1) White (2) Green (3) Yellow (4) Orange (5) Pink (6) Red (7) Purple
6	Petal shape	(1) Linear (2) Oblong (3) Oval (4) Diamond (5) Obovate (6) Spatulate
7	Petal spot	(1) None (2) Yes
8	Petal stripes	(1) None (2) Yes
9	Middle labellum primary color	(1) White (2) Green (3) Yellow (4) Orange (5) Pink (6) Red (7) Purple
10	Labellum spot	(1) None (2) Yes
11	Labellum stripes	(1) None (2) Yes
12	The shape of the middle lobe of the labellum	(1) Narrow triangle (2) Triangle (3) Trapezoid (4) Round (5) Oblate
13	Trisomy of the Labellum	(1) None (2) Yes
14	Size of Pseudobulb	(1) Hardly any (2) Not obvious or small (3) Obvious
15	Leaf edge	(1) whole edge (2) fine serration (3) coarse serration
16	Leaf tip traits	(1) Sharp (2) Obtuse (3) Concave
